# Owner-Directed Feline Aggression in Thailand: Characteristics, Associated Factors, and a Clinical Comparison of Treatments

**DOI:** 10.3390/life16020307

**Published:** 2026-02-10

**Authors:** Jarawee Supanta, Worakan Boonhoh, Orachun Hayakijkosol, Tuempong Wongtawan

**Affiliations:** 1 Akkhraratchakumari Veterinary College, Walailak University, Nakhon Si Thammarat 80160, Thailand; jarawee.su@wu.ac.th (J.S.); worakan.bo@mail.wu.ac.th (W.B.); 2 Centre for One Health Research Centre, Walailak University, Nakhon Si Thammarat 80160, Thailand; 3 Veterinary Preclinical Science, College of Science and Engineering, Academy Division, James Cook University, Townsville, QLD 4811, Australia; orachun.hayakijkosol1@jcu.edu.au; 4 Pet Mental and Behaviour Clinic, Walailak University’s Animal Hospital, Nakhon Si Thammarat 80160, Thailand

**Keywords:** aggressive, bite injury, cannabidiol, cat, fluoxetine

## Abstract

Despite the global increase in cat ownership, some cats exhibit owner-directed aggression, resulting in caregiver injury, infection, and anxiety. Severe cases are commonly treated with selective serotonin reuptake inhibitors such as fluoxetine; however, adverse effects, particularly transient anorexia, often discourage treatment initiation. Cannabidiol (CBD), a natural compound with reported anxiolytic properties and minimal anorexic effects, may represent an alternative therapy. This study aimed to characterise owner-directed feline aggression in Thailand, identify associated factors, and compare the efficacy of CBD with fluoxetine. Most caregivers were females aged 20–40 years, and most cats were neutered mixed-breeds aged 1–6 years living indoors in multi-human and multi-cat households. For demographic variables, only human–cat interactions (e.g., petting) were significantly associated with aggression. Handling-induced aggression was universal, with grooming as the most common trigger (56%). In a single-blind, 4–8-week trial, 100 cats were randomly assigned to control, CBD 1 mg/kg/day, CBD 2 mg/kg/day, fluoxetine 0.5–1 mg/kg/day, or combined CBD and fluoxetine. Aggression scores decreased significantly in all treatment groups compared with control (*p* < 0.05), with no differences among active treatments. CBD at 1 mg/kg/day showed efficacy comparable to fluoxetine without anorexic effects.

## 1. Introduction

Cat ownership has become increasingly popular worldwide, with ownership rates continuing to rise [1]. Caring for cats can contribute to a lower risk of cardiovascular diseases and mental disorders, potentially through increased physical activity and reduced loneliness among caregiver [2,3]. However, inappropriate care and management of cats may result in behaviour problems [4], leading to negative impacts on caregivers’ physical health, including biting injuries and zoonotic diseases [5,6] and may also be associated with psychological disorders such as depression and schizophrenia [7,8].

In this manuscript, the term “caregiver” is used in preference to “owner” to reflect the human–cat relationship; however, the term “owner-directed aggression” is retained where it refers specifically to the established behavioural classification used in the veterinary literature.

Owner-directed aggression by cats is considered one of the most problematic behavioural issues [9]. This behaviour is multifactorial, influenced by feline characteristics (e.g., sex and health status), the home environment (e.g., numbers of cat and arousal level), and human–cat interactions (e.g., training methods, owner responses, and petting) [9,10,11]. Aggressive behaviour has been significantly associated with both physical disorders (e.g., FeLV/FIV infection and hyperandrogenism) and early-life psychological trauma, including experiences such as being chased, handled roughly by children, falling from a height, or involvement in a road traffic accident [10,11]. Higher levels of aggression have been reported in female cats and in cats living in single-cat households compared with male cats and those housed in multi-cat households [9,11]. In addition, aversive owner responses (e.g., verbal reprimands, loud noises, holding or avoiding the cat) were consistently associated with increased frequency and severity of aggression toward people and other animals, whereas training enrichment appeared protective [9,11]. Human–cat interactions, particularly petting and play, have also been identified as common contexts in which aggressive responses occur, which may arise from either excessive handling or individual sensitivity to touch [1,11].

Owner-directed aggression has important implications for both feline welfare and the quality of the human–animal bond [12,13]. Beyond the immediate risk of physical injury to caregivers, aggressive behaviour in cats may increase the likelihood of exposure to zoonotic pathogens, including rabies virus, *Bartonella henselae*, and *Sporothrix globosa* [14,15,16,17]. Such incidents can also undermine trust and emotional attachment between cats and their owners, leading to breakdowns in the human–animal relationship. In severe or persistent cases, feline aggression may contribute to caregivers’ frustration, relinquishment, or abandonment of cats, thereby exacerbating broader animal welfare and public health concerns [18,19].

Treatment for feline aggression involves many steps, including behaviour modification for the caregivers and cats, as well as medicine in cases of severe behaviour [20]. From a neurophysiology perspective, the aggression has been associated with a reduction in calming neurotransmitters such as serotonin [21]. The use of selective serotonin reuptake inhibitors (SSRIs), such as fluoxetine, can increase the level of serotonin in the nervous system, resulting in reduced aggression in dogs and cats [22,23,24,25]. However, this drug has common side effects, including transient anorexia, sleepiness, vomiting and diarrhoea (1–14 days); the rare side effects include temporary irritability, ataxia, and urinary retention [26,27,28].

Recently, natural products such as hemp oil containing cannabidiol (CBD) have been investigated for reducing stress and anxiety in cats during transport, handling, and separation, with two studies reporting beneficial effects [29,30]. In contrast, another study found that CBD did not reduce stress responses associated with transport or exposure to unfamiliar individuals in cats [31]. The reduction of stress and anxiety may be associated with the sedation effect of CBD [30,32], triggering inhibiting (calming) neurotransmitters (e.g., GABA and serotonin) and anti-inflammatory and analgesic properties via cannabinoid receptors 1 and 2 [33,34,35]. In contrast to SSRIs, CBD seems to stimulate appetite rather than anorexia in some dogs and cats [36,37].

In this study, we hypothesised that cannabidiol (CBD) could serve as an alternative treatment for reducing feline aggression through its analgesic and anti-inflammatory properties, as well as its modulatory effects on the serotonin and GABAergic systems that promote calming. In addition, CBD may mitigate adverse effects associated with fluoxetine when used in combination. The objectives of this study were to: (1) characterise owner-directed aggression in cats in Thailand; (2) identify associated factors and triggering events; and (3) evaluate the efficacy and adverse effects of CBD in the management of feline owner-directed aggression compared with conventional treatment using fluoxetine.

## 2. Materials and Methods

### 2.1. Aggression Score of Owner-Directed Feline Aggression

In this study, a composite aggression score was used to quantitatively evaluate treatment effects. The owner-directed feline aggression score was calculated as the average score of an aggressive behaviour scale (Table 1) across 8 common aggression-triggering events (Table 2) for each cat. For each event, caregivers rated their cat’s aggressive behaviour on a 5-point scale ranging from 0 (no aggression) to 4 (very aggressive, e.g., frequent (weekly) biting or scratching).

The aggression score and 8 common triggered events were already validated in our previous national survey involving approximately 3000 participants [1]. Internal consistency of the composite aggression score was high, with Cronbach’s alpha and McDonald’s ω both equal to 0.88. Principal component analysis (PCA) with varimax rotation supported a single-factor structure (factor loadings = 0.61–0.80), indicating that aggressive responses across owner-related contexts—including handling, fear, play, and possessive situations—represent a unified construct of owner-directed (situational) feline aggression.

### 2.2. Recruitment Process

Caregivers who reported being bitten or scratched by their cats on a frequent basis (at least weekly) were invited to participate in this research project at Walailak University Animal Hospital through the WU Small Animal Hospital notice board, social media platforms, and website. Inclusion criteria required a human caregiver aged ≥20 years and a cat aged ≥6 months with no current physical illness. Eligible cats must not have previously been prescribed or administered any psychoactive medications (e.g., fluoxetine, sertraline, or gabapentin) or behavioural supplements (e.g., CBD or GABA).

Cats and caregivers who initially met the inclusion criteria were invited to participate in a veterinary-led interview and to complete a questionnaire to classify the type of aggression and to assess aggression scores. Cats exhibiting high aggression scores (>2) were subsequently selected for inclusion in the study. Only one cat per household was permitted to participate. Of approximately 200 applications received, 100 met the eligibility criteria and were enrolled in the study.

All cats meeting the inclusion criteria (n = 100) were enrolled and included in baseline data collection and analysis. Exclusion criteria were applied only after enrolment during the treatment and follow-up period. Cats were withdrawn from the study if they developed intercurrent illness, if caregivers were unable to comply with the study protocol, or if caregivers failed to report behavioural changes or attend scheduled follow-up appointments.

### 2.3. Diagnosis of Owner-Directed Feline Aggression

The diagnosis included a physical examination and a blood evaluation to rule out physical diseases related to behavioural problems [1]. The caregiver was constructively interviewed by a veterinary behaviourist to diagnose owner-directed aggression and identify the possible type and cause of aggression. All the caregivers provided videos that demonstrated aggressive behaviour in their cats.

### 2.4. Demographic Data

Demographic data was answered by the caregiver using a demographic questionnaire (Supplementary Data, Table S1) that collected information on the caregivers (e.g., gender, age, and experience with cat care), the cats (e.g., breed, age, sex, and neuter status), the environment (e.g., house type, living area, number of cats and humans, and the presence of children or other animals), management (e.g., food, vaccines, and enrichments), and interaction between caregivers and cats (e.g., play, sleep, pet, hug, kiss, and company).

### 2.5. Experimental Design

Aggressive cats were randomly allocated to 5 groups in a single-blind trial, in which caregivers were blinded to treatment allocation. The groups comprised a control group (CON), which received one multivitamin (including vitamin C and E, Zinc and Omega 3) tablet (Dr.ChoiceDerma-B^TM^, Interpharma, Bangkok, Thailand) once daily as a placebo; a cannabidiol group receiving CBD oil (8% CBD; DR.CBD Ltd., Bangkok, Thailand) at a dose of 1 mg/kg/day (CBD1); a second CBD group receiving 2 mg/kg/day (CBD2); a fluoxetine group receiving fluoxetine tablets at 0.5–1 mg/kg/day (FLU); and a combination group receiving CBD at 1 mg/kg/day plus fluoxetine at 0.5–1 mg/kg/day (CBD1 + FLU). To maintain blinding, caregivers were not informed of the drug names and were told only that their cats received “Drug A”, “Drug B”, or “Drug C”. Study medications were dispensed without original packaging or labels, and no written or verbal information identifying the drugs was provided. While differences in formulation (oil versus tablet) could have allowed potential unblinding, caregivers were not informed of the drug identities.

Caregivers in all groups received education on feline behaviour and behavioural modification advice from a veterinarian. Treatments were administered for a minimum of 4 weeks. Comprehensive behavioural assessments, including caregiver interviews, questionnaires, and aggression score evaluations, were conducted at baseline and at four weeks of treatment. In addition, caregivers reported daily medication compliance and brief behavioural observations via the LINE online application (LY Corporation, Tokyo, Japan). Cats that continued to exhibit residual aggressive behaviours (e.g., occasional biting or scratching) after four weeks of treatment, despite a reduction in overall aggression scores and attack frequency, continued on the same assigned medication without dose escalation or drug substitution until behavioural resolution was achieved. These cases were typically cats with very high baseline aggression scores.

### 2.6. Statistical Analysis

Statistical analyses were performed using Jamovi software (version 2.6.26). The Wilcoxon signed-rank test was used to compare differences before and after treatment for non-parametric data. The Kruskal–Wallis test (H) followed by Dwass–Steel–Critchlow–Fligner pairwise comparisons (DSCF) were applied to compare differences among treatment groups. Fisher’s exact test (FET) was used to assess differences in side effects. The *p* value less than 0.05 was considered a statistically significant differences for H, DSCF, and FETs.

To identify the correlation among aggression triggers, Spearman’s rank correlation test (ρ) was used to identify the correlation between types of aggression. Multiple pairwise correlations were assessed; therefore, *p*-values were adjusted using the Bonferroni correction (the number of unique pairwise correlations were 28 comparisons); therefore, *p* < 0.0018 (α = 0.05/28 = 0.0018) were considered statistically significant.

Additionally, association between demographic factors and aggression score was determined by the Man-Whitney U test (2 categories) and Kruskal–Wallis (>2 categories). The *p* value of less than 0.05 was considered statistically correlated.

## 3. Results

A total of 100 cats and their caregivers were enrolled and included in baseline descriptive analyses and identify assocaited factors with feline aggression (Section 3.1, Section 3.2, Section 3.3, Section 3.4, Section 3.5, Section 3.6, Section 3.7, Section 3.8 and Section 3.9). During the study period, a subset of participants was withdrawn according to predefined post-enrolment exclusion criteria. Overall and group-specific withdrawal numbers and reasons are summarised below and described in detail in Section 3.10 (effect of treatments).

### 3.1. Caregiver Demographics

A total of 100 people participated as caregivers in the study. Caregiver characteristics are shown in Table S2. The majority of caregivers were women, accounting for 80.00% (n = 80), aged 20–40 years old (68.00%, n = 68). For the experience, 55.00% (n = 55) had experience for ownership less than 5 years, the remaining 45.00% had more than 5 years of experience.

### 3.2. Cat Demographics

Cat characteristics are shown in Table S3. The cat population was nearly evenly split by sex: 51 female cats (51.00%) and 49 male cats (49.00%). The predominant age group for cats was 1–6 years old (91.00%, n = 91). Regarding breed, the mixed breed was the most populated at 74.00% (n = 74). For the reproductive status, most of the cats were neutered (66.00%, n = 66).

### 3.3. Environmental Characteristics

Environmental characteristics are shown in Table S4. The majority of cats lived in houses (80.00%, n = 80), while the remainder resided in condominiums. Most were primarily kept indoors (74.00%, n = 74), and others were semi-indoor cats. Most cats lived with 2–4 humans (67.00%, n = 67), had no children in the household (76.00%, n = 76), cohabited with other cats (65.00%, n = 65), and lived without other animal species (65.00%, n = 65).

### 3.4. Cat Management by Caregivers

Cat management practices are presented in Table S5. Most cats were fed commercial food (95.00%, n = 95), with food available throughout the day (57.00%, n = 57), and had water provided in a bowl (74.00%, n = 74). They were annually vaccinated (60.00%, n = 60), had an optimal body condition score (61.00%, n = 61), and had a litter box equal to the number of cats in the household (62.00%, n = 62). Most caregivers also provided environmental enrichment for their cats, including scratchers (82.00%, n = 82), and climbing apparatuses (55.00%, n = 55).

### 3.5. Human–Cat Interaction

A detailed human–cat interactions are shown in Table S6. Caregivers commonly used reward-based (78.00%, n = 78) rather than punishment-based training (23.00%, n = 23). They often hug/hold (69.00%, n = 69), pet their head/chin (75.00%, n = 75), pet their abdomen (68.00%, n = 68), and kiss their cats (58.00%, n = 58). Most owner often sleep with cats (63.00%, n = 63), and company with cat when feeding (53.00%). Around 54.00% (n = 54) of caregivers left a cat alone at home more than 4 h daily, while 23.00% of caregivers often travelled with a cat.

### 3.6. Associatiomn of Demographic Factors with Aggression Score

From 100 cats, the median aggression score was 2.37 (IQR = 0.65), ranging from 1.75 to 4.00. Most evaluated factors were not statistically associated (*p* ≥ 0.05) with aggression score, and the detail is in Tables S2–S6. However, several variables showed significant associations. Purebred cats had higher aggression scores than mixed-breed cats (*p* = 0.02). Cats that were frequently exposed to reward-based training exhibited lower aggression scores (*p* = 0.03). In addition, several forms of human–cat interaction—including hugging (*p* = 0.03), head and chin petting, abdominal petting (*p* = 0.02), and kissing (*p* = 0.004)—were significantly associated with aggression score, with more frequent interaction corresponding to lower aggression levels.

### 3.7. Situation That Triggered Severe Owner-Directed Aggression

All situations triggering owner-directed aggression are presented in Table 3. The most common trigger for severe feline aggression (defined as an aggression scale score of 4 in at least one event), characterised by frequent biting and scratching, was the caregiver’s attempts to bathe, groom, brush, or trim the cat’s nails (56.00%). This was followed by being scolded, hit, or threatened with physical punishment (34.00%); play interactions with the caregiver (32.00%); and being held, hugged, or kissed (29.00%). Notably, during play interactions, all caregivers reported using bare hands, often allowing biting behaviours from kittenhood into adulthood.

### 3.8. Correlation Among Events That Trigger Aggression

The statistical correlations among aggression-triggering events are presented in Table 4. Multiple pairwise comparisons were conducted using Spearman’s rank correlation with Bonferroni correction (adjusted α = 0.0018). After adjustment, strong associations remained between touching/petting and hugging/kissing, and between food- or toy-triggered aggression and caregiver movement and territorial entry (*p* < 0.001), indicating that aggression occurring in these paired contexts tended to co-occur within the same individuals.

Most pairwise correlations showed low correlation coefficients (Spearman’s ρ < 0.30), reflecting limited overlap among different trigger contexts. Several weak correlations were observed among handling-, arousal-, and territorial-related events (ρ = 0.20–0.30); however, these were not statistically significant after Bonferroni correction. Some associations approached the adjusted significance threshold, including the correlation between touching/petting and play-related aggression (ρ = 0.30, *p* = 0.002). Overall, the predominance of small effect sizes suggests that aggressive responses were generally specific to particular triggering situations rather than broadly shared across contexts.

### 3.9. Occurrence of Aggression Types

Cat’s aggressive behaviour was classified into 4 categories of aggression. Handling-induced aggression (triggered by interactions such as petting, grooming, kissing, or being held) was the most prevalent type and was observed in all cats (100%, n = 100). This was followed by play-induced aggression (e.g., during play or predatory behaviour, including attacks when the owner moved) in 78% of cats. Fear-induced aggression, triggered by noise or human responses such as reacting to or punishing the cat, was observed in 38% of cases, while possessive aggression (associated with guarding food, toys, or territory) was also present in 38% of cats.

Multiple aggression types commonly co-occurred within individuals. Overall, 28.00% of cats exhibited all four aggression types, 44.00% displayed three types, 19.00% displayed two types, and 9.00% exhibited only a single type of aggression.

### 3.10. Effect of the CBD and Fluoxetine on Feline Owner-Directed Aggression

Of the 100 cats initially recruited, several were excluded from the study due to caregivers’ non-compliance with the 4–8-week protocol, such as irregular medication administration, failure to provide daily behavioural reports, or failure to attend scheduled veterinarian appointments. The numbers of cats remaining in each group are shown in Table 5: 14 in the control group, 14 in the CBD1 group, 13 in the CBD2 group, 12 in the FLU group, and 14 in the CBD1 + FLU group. Comparisons of caregiver-directed aggression before and after treatment, as well as among the experimental groups, are detailed in Table 5.

At baseline (before treatment), cats in all groups displayed high median aggression scores toward their caregivers, ranging from 2.38 to 3.04 (Table 5), with no significant differences among groups before treatment (X^2^ = 7.35, df = 4, *p* = 0.11). In contrast, treatment outcomes (cat behaviours after treatment) differed significantly across groups (X^2^ = 23.91, df = 4, *p* < 0.0001).

Within-group comparisons demonstrated a significant reduction in caregiver-directed aggression following treatment across all intervention groups (*p* ≤ 0.002). After treatment, cats in the control group exhibited significantly higher aggression scores than those in all treatment groups (*p* < 0.001). No significant differences were observed among the CBD1, CBD2, FLU, and CBD1 + FLU groups (*p* ≥ 0.05); however, the CBD1 + FLU group showed the greatest median reduction in aggression scores.

Based on caregiver observations, a reduction in aggressive behaviour was evident within a week in cats treated with CBD alone or in combination with fluoxetine, whereas cats receiving fluoxetine monotherapy showed a noticeable decrease in aggression within 2 weeks of treatment.

By week 4, the majority of cats in the treatment groups (CBD and FLU) (73.58%, 39/53) exhibited no aggressive behaviours (biting or scratching), allowing the discontinuation of fluoxetine and CBD. Although aggression scores decreased in all cats, 16 individuals continued to display aggressive behaviours and therefore received an additional 4 weeks of treatment; by the end of this extended period, none of the remaining cats showed aggressive behaviour.

At the 4-week assessment, the CBD1 + FLU group had the lowest proportion of cats exhibiting persistent aggressive behaviour (21.42%, n = 3/14), followed by CBD2 (23.07%, n = 3/13), FLU (41.66%, n = 5/12), and CBD1 (42.85%, n = 6/14). However, no significant difference was detected among treatment groups (χ^2^(3) = 2.28, *p* = 0.52).

### 3.11. Effect of CBD and Fluoxetine on Grooming-Induced Aggression

Grooming was identified as the most frequent trigger of aggressive behaviour in this study, prompting further investigation into the effects of treatment on grooming-induced aggression as shown in Table 6. After treatment, a statistically significant reduction in aggression was observed in all groups, including the control group. Prior to treatment, no significant differences were detected among the treatment groups. In contrast, post-treatment analysis revealed that the CBD1, CBD2, and CBD + FLU groups showed significantly lower aggression scores compared with the control group (*p* < 0.05), while no significant differences were found among the three treatment groups (*p* ≥ 0.05).

### 3.12. Side Effects of CBD and Fluoxetine

Fluoxetine treatment was associated with sleepiness in 83.33% of cats (n = 10/12) and transient mild-to-moderate anorexia in 75.00% (n = 9/12), both occurring for less than one week; however, these adverse effects caused considerable concern among caregivers. In contrast, CBD treatment induced sleepiness in 57.14% of cats in the CBD1 group (n = 8/14) and 61.53% in the CBD2 group (n = 8/13), without evidence of anorexia. Notably, increased appetite was observed in 35.71% of cats in the CBD1 group (5/14) and 53.84% in the CBD2 group (n = 7/13). In the combined CBD+FLU group, anorexia was not observed, although sleepiness occurred in all cats.

## 4. Discussion

The present study demonstrated that CBD at a dose of 1 mg/kg/day significantly reduced owner-directed aggression in cats, with caregivers reporting perceived improvement within days. Increasing the dose to 2 mg/kg/day did not result in a greater reduction in aggression scores compared with the 1 mg/kg/day dose; however, it appeared to accelerate the onset of clinical response and was associated with a higher frequency of increased appetite and sleepiness. Moreover, the combination of CBD and fluoxetine appeared to offer greater benefit than the other treatment groups, with a tendency toward a faster therapeutic response. This combination was not associated with alterations in appetite-related behaviour; however, cats in this group appeared to exhibit increased sleepiness compared with those receiving other treatments.

However, the minimal effective dose remains unknown, as lower doses such as 0.5 mg/kg/day were not evaluated in this study. Moreover, CBD produced results comparable to fluoxetine (conventional treatment) but was associated with fewer side effects, causing temporary sleepiness but not anorexia. Notably, unlike fluoxetine, CBD in this study did not suppress appetite; instead, it appeared to stimulate it. Similar to the current study, the appetite-stimulating effect of CBD has been previously observed in some cats and dogs [36,37]. Conversely, in humans there is moderate evidence suggesting that CBD tends to reduce appetite and body weight [38,39].

Research on the effects of CBD on aggression remains limited. To our knowledge, this study is the first to demonstrate the effectiveness of CBD in reducing aggressive behaviours in cats. Similar findings have been reported in mice, where CBD was shown to reduce aggression [40]. In dogs, however, the evidence is inconsistent: some studies suggest that CBD may reduce aggressive behaviour [40,41], whereas others have reported no significant effects [42]. Consistent with this variability, our preliminary clinical observations indicate that aggressive dogs may show limited responsiveness to CBD (Wongtawan, unpublished data).

The mechanism of CBD to reduce aggression may be related to its manipulation of neurotransmitters [30,31,32,33,34,35], although research on the effects of CBD on neurotransmitters remains limited. The initial study in cat aggression has demonstrated that the hypothalamus plays a key role in regulating aggressive behaviour, specifically through regions known as the hypothalamic attack area [43], which are also conserved in many species [44]. In the hypothalamus, several neurotransmitters have been believed to moderate aggressive behaviour, particularly GABA and serotonin [44,45]. Some studies in rats, mice, cats, dogs, monkeys, and humans have revealed that low levels of GABA or serotonin in the brain or blood is associated with increased aggression [44,46,47,48,49] and administration of SSRIs has been shown to increase serotonin levels and reduce aggression in both dogs and cats [22,23,25]. Previous studies in mice and rats have shown that CBD exerts antidepressant-like effects, likely through the modulation of serotonin production and secretion; however, these effects appear to develop with chronic rather than acute administration [50,51,52]. In contrast, our study revealed that aggressive cats seemed highly sensitive to CBD, showing noticeable behavioural improvements within a single night. Such interspecies variation in neurotransmitter sensitivity and responsiveness may explain these differences, as our clinical observations suggest that CBD exerts less behavioural impact in dogs (Wongtawan, unpublished data). Regarding GABAergic mechanisms, CBD has been shown to bind to GABA receptors in Xenopus and human tissue studies [53,54] and to positively modulate the glutamate-GABA system in healthy humans, which may contribute to its calming effect [55].

In this study, a reduction in aggressive behaviour was observed within the first two weeks in cats treated with CBD, fluoxetine, or their combination, which is earlier than the typical onset of anxiolytic efficacy reported for selective serotonin reuptake inhibitors (SSRIs), generally occurring after 2–3 weeks of treatment [26]. This early behavioural improvement may therefore not fully reflect the established anxiolytic mechanism of these medications. Possible contributing factors include transient behavioural suppression or mild sedation during treatment initiation. Further controlled longitudinal studies are needed to clarify the temporal profile and underlying mechanisms of treatment-associated behavioural modification in cats.

Grooming was the most common trigger-induced aggression in this study; however, no prior research has specifically examined this form of aggression in cats. This type of aggression may be influenced by multiple factors, including pain, stress, fear, discomfort, or overstimulation (touch-sensitive) during grooming [56]. Furthermore, petting cats, such as touching, hugging, and kissing, was also a common trigger in the present study, which is associated with the tendency of Thai caregivers to frequently pet, hug, and kiss their cats [1], behaviours that may increase the risk of bites or scratches. Similar to a previous study, over 40% of cats display aggression toward their caregiver during excessive petting [9]. Collectively, touch-, petting-, and grooming-induced aggression can be categorised as handling aggression which has been the most common type of feline aggression in both the current and previous studies [12]. To minimise handling-related aggression, caregivers should be educated to avoid unnecessary physical contact with their cats and to apply stress-free handling methods combined with reward-based reinforcement to help reduce stress during interactions [57]. In particularly fearful cats, pharmacological interventions such as gabapentin or diazepam may be required to alleviate fear and aggression, thereby minimising the risk of injury to handlers [35,56,57]. Another common trigger for aggression observed in this study was a play interaction between humans and cats. All participating caregivers reported using their bare hands or feet during play, allowing cats to bite or chew from kittenhood into adulthood. As a result, some cats may develop a habit to play-biting and begin to associate human hands and feet with toys, continuing this behaviour even when not permitted. Encouraging play with appropriate toys or alternative objects, rather than human body parts, may help reduce this undesired behaviour [58].

In this study, behaviour modification strategies were provided to caregivers by veterinarians, and most caregivers attempted to adhere to these guidelines. This resulted in a modest improvement in feline aggression, as observed in the control group; however, the effect was inferior to that achieved when behaviour modification was combined with pharmacological intervention. These findings suggest that behaviour modification alone may provide limited benefit and may be more suitable for managing mild aggression, whereas cases of severe aggression, particularly those involving biting, are likely to require pharmacological treatment.

Additional limitations of this study include the absence of data on the precise minimum effective dose of CBD and the lack of information regarding its long-term side effects, which should be addressed in future research. Previous studies in laboratory animals and humans have indicated that excessive or prolonged exposure to CBD may increase the risk of toxicity in the liver, reproductive, nervous, and digestive systems [59]. Moreover, full behavioural assessment over periods longer than one week may be necessary to confirm the stability of treatment effects, as fluoxetine typically requires at least 4–8 weeks to achieve optimal efficacy. Based on the current study, which included a large clinical sample of cats with aggression, a four-week course of CBD appeared to be sufficient for most cats, with no caregivers reporting recurrence of aggressive behaviour during the two-month follow-up period. Although many caregivers in this study reported rapid behavioural improvement, longer-term evaluation remains important issue.

In this study, most demographic variables were not significant associated with aggression scores. This may be attributable to the inclusion of only cats exhibiting severe aggressive behaviour (high aggression score), characterised by high aggression scores and frequent biting or scratching. To identify associated or risk factors more effectively, future studies should include cats with a wider range of aggression severity, including none, mild or moderate aggression. Nevertheless, few significant associations were observed in relation to human–cat interactions, including holding, hugging, petting, and kissing. Cats whose guardians interacted with them more frequently exhibited lower aggression scores compared with cats whose guardians interacted with them less often. Due to the cross-sectional design of this study, the direction of the association between human–cat interaction and feline aggression cannot be determined. While frequent interaction may contribute to reduced aggression, it is also possible that guardians of highly aggressive cats intentionally limit physical contact to avoid bite or scratch injuries. Longitudinal or interventional studies are therefore required to clarify causality. Future research should include cats across a broader range of aggression severity and incorporate prospective follow-up, structured interaction interventions, and explicit assessment of guardian avoidance behaviours.

## 5. Conclusions

This study suggests that handling-induced aggression, primarily triggered by grooming, is the most common form of owner-directed feline aggression in cats in Thailand. Breed and human–cat interactions including petting, hugging, and kissing are associated with feline aggression. Oral administration of CBD at a dose of 1 mg/kg/day effectively reduced owner-directed aggression, with efficacy comparable to fluoxetine. Unlike fluoxetine, CBD was not associated with anorectic side effects and instead appeared to stimulate appetite. Furthermore, the combination of CBD and fluoxetine may offer the advantage of a faster treatment response without inducing anorexia.

## Figures and Tables

**Table 1:** Scale of feline aggressive behaviour as rated by their owners. The scale was rated for each event (as detailed in Table 2) that might trigger aggressive behaviours in cats.

Aggressive Behaviour Scale	Description of Aggressive Behaviour Toward the Caregivers
0 (None)	Never observed aggressive behaviours such as growling, piloerection, swatting, scratching, or biting.
1	Rarely (>3 months) observed mild/moderate aggressive behaviours, such as swatting, growling, or slight piloerection, but never biting or scratching.
2	Occasionally (monthly) observed mild/moderate aggressive behaviour, such as swatting, growling, or slight piloerection, and rarely displayed severe aggressive behaviour, such as scratching or biting.
3	Frequently (weekly) observed, mild/moderate aggressive behaviours such as swatting, growling, or slight piloerection, and occasionally (monthly) displayed severe aggressive behaviour such as scratching or biting.
4 (Very aggressive)	Often (daily to weekly) displays very aggressive behaviours, including scratching or biting.

**Table 2:** Questionnaire related to events that trigger aggression.

Aggressive Behaviour Scale	Events That May Trigger Cat Aggression
0–4	1. When the cat is touched or petted by the caregiver (Touch/Pet)
0–4	2. When being held, hugged, or kissed by the caregiver (Hug/Kiss)
0–4	3. When eating food or playing with toys and the caregiver approaches (Food/Toy)
0–4	4. When playing with the caregiver, either with toys or bare hands (Play)
0–4	5. When people in the house walk or move around (Move)
0–4	6. When frightened by scolding, being hit, threatened, or exposed to loud noises (Frighten)
0–4	7. When bathed, groomed, brushed, or having nails trimmed by the caregiver (Groom)
0–4	8. When the caregiver enters to the area where the cat is resting (Territory)

**Table 3:** Severe aggressive behaviour (aggressive behaviour scale = 4, weekly attack a caregiver) toward a caregiver displayed during each event (number of cats = 100).

Events That Trigger Cat Aggressive Behaviour	Cats Displayed Severe Aggression	
	Number of Cats	Percentage
1. When touched or petted by the caregiver	26	26.00
2. When held, hugged, or kissed by the caregiver	29	29.00
3. When eating food or playing with toys and the caregiver approaches	14	14.00
4. When playing with the caregiver, either with toys or bare hands	32	32.00
5. When people in the house walk or move around	16	16.00
6. When frightened by scolding, being hit, threatened, or exposed to loud noises.	34	34.00
7. When bathed, groomed, brushed, or having nails trimmed by the caregiver	56	56.00
8. When the caregiver enters to the area where the cat is resting	11	11.00

**Table 4:** Correlation among aggression-triggering events (Spearman’s rank correlation coefficient with Bonferroni correction).

Events		1. Touch/Pet	2. Kiss/Hug	3. Food/Toy	4. Play	5. Movement	6. Frighten	7. Groom
2. Kiss/Hug	ρ	0.50	—	—	—	—	—	—
	df	98	—	—	—	—	—	—
	*p* value	<0.001 *	—	—	—	—	—	—
3. Food/toy	ρ	0.16	0.18	—	—	—	—	—
	df	98	98	—	—	—	—	—
	*p* value	0.10	0.06	—	—	—	—	—
4. Play	ρ	0.30	0.18	0.13	—	—	—	—
	df	98	98	98	—	—	—	—
	*p* value	0.002	0.07	0.19	—	—	—	—
5. Movement	ρ	−0.002	−0.12	0.32	0.16	—	—	—
	df	98	98	98	98	—	—	—
	*p* value	0.98	0.20	<0.001 *	0.09	—	—	—
6. Frightened	ρ	0.22	0.03	−0.01	0.27	0.20	—	—
	df	98	98	98	98	98	—	—
	*p* value	0.02 *	0.75	0.86	0.006 *	0.04 *	—	—
7. Groom	ρ	0.21	0.11	0.11	0.16	−0.16	0.09	—
	df	98	98	98	98	98	98	—
	*p* value	0.03	0.23	0.24	0.10	0.09	0.37	—
8. Enter its territory	ρ	0.22	0.30	0.34	0.03	0.21	0.25	0.10
	df	98	98	98	98	98	98	98
	*p* value	0.02	0.002	<0.001 *	0.70	0.03	0.009	0.30

* Represents a significant correlation between 2 events after Bonferroni correction (*p* < 0.0018). ρ is Spearman’s rho value. DF is degree of freedom.

**Table 5:** Owner-directed aggression was compared before and after treatment.

Groups	No. of Cats	Median of Aggression Score (IQR)		Statistics (W)	*p* Value	Median Difference	Effect Size
		Before	After				
Control	14	2.88 (0.84)	2.60 (0.77) ^a^		0.001 *	0.28	1
CBD1	14	2.66 (1.24)	1.06 (1.18) ^b^	105.00	<0.001 *	1.60	1
CBD2	13	2.38 (0.75)	1.00 (0.67) ^b^	76.00	0.002 *	1.38	1
Flu	12	2.40 (0.88)	1.17 (1.05) ^b^	105.00	<0.001 *	1.23	1
CBD1 + FLU	14	3.04 (0.70)	0.93 (0.53) ^b^	78.00	<0.001 *	2.11	1
Statistics (H)		5.82	0.45				
*p* value		0.12	<0.001				

* Represents statistically significant difference (*p* < 0.05) between before and after treatment (row) by Wilcoxon signed-rank test (W). ^a,b^ Represents a statistically significant difference (*p* < 0.05) between groups by multiple comparison using the Dwass–Steel–Critchlow–Fligner test.

**Table 6:** Grooming-induced aggression was compared before and after treatment.

Groups	No. of Cats	Median of Aggression Score (IQR)		Statistics (W)	*p* Value	Median Difference	Effect Size
		Before	After				
Control	13	4.00 (1.00)	3.00 ^a^ (0.00)	45.00	0.005 *	1.00	1
CBD1	13	3.00 (2.00)	2.00 ^b^ (1.00)	55.00	0.005 *	2.00	1
CBD2	13	3.00 (2.00)	1.00 ^b^ (1.00)	55.00	0.005 *	2.00	1
Flu	12	4.00 (1.00)	2.00 ^a^ (3.00)	66.00	0.003 *	2.00	1
CBD1 + FLU	12	3.50 (1.00)	1.00 ^b^ (0.50)	78.00	0.002 *	1.50	1
Statistics (H)		6.11	13.90				
*p* value		0.19	0.007				

* Represents statistically significant difference (*p* < 0.05) between before and after treatment (row) by Wilcoxon signed-rank test (W). ^a,b^ Represents a statistically significant difference (*p* < 0.05) between groups by multiple comparison using the Dwass–Steel–Critchlow–Fligner test.

## Data Availability

The original data presented in the study are openly available to download at https://data.mendeley.com/datasets/t7wxvjfyct/1 (accessed on 5 February 2026).

## Supplementary Materials

The following supporting information can be downloaded at: https://www.mdpi.com/article/10.3390/life16020307/s1. The supplementary materials include detailed information on the demographic questionnaire (Table S1) and the associations between demographic variables and aggression score, encompassing caregiver characteristics (Table S2), cat characteristics (Table S3), environmental characteristics (Table S4), cat management practices (Table S5), and human–cat interactions (Table S6).

## Abbreviations

The following abbreviations are used in this manuscript:

CBD	cannabidiol
FLU	fluoxetine

## References

[1] Boonhoh W., Poolkhet C., Sriphavatsarakom P., Waran N., Wongtawan T. (2025). Cat Ownership and Cat–Owner Interaction in Thailand. Anthrozoos.

[2] Ravenscroft S., Barcelos A.M., Mills D. (2021). Cat-Human Related Activities Associated with Human Well-Being. Hum. Anim. Interact. Bull..

[3] Watson K.M., Kahe K., Shier T.A., Li M. (2023). Age Modifies the Association between Pet Ownership and Cardiovascular Disease. Front. Vet. Sci..

[4] Amat M., Torre J., Fatjó J., Mariotti V., Wijk S., Manteca X. (2009). Potential Risk Factors Associated with Feline Behaviour Problems. Appl. Anim. Behav. Sci..

[5] Kheiran A., Palial V., Rollett R., Wildin C.J., Chatterji U., Singh H.P. (2019). Cat Bite: An Injury Not to Underestimate. J. Plast. Surg. Hand Surg..

[6] Palacio J., León-Artozqui M., Pastor-Villalba E., Carrera-Martín F., García-Belenguer S. (2007). Incidence of and Risk Factors for Cat Bites: A First Step in Prevention and Treatment of Feline Aggression. J. Feline Med. Surg..

[7] Huang Y., Qing H., Pan Y., Wei Y., Zhang K., Song J., Wang C., Dong H., Tang S. (2026). Exploring the Dual Effects of Pet Ownership on Mental Health Outcomes in Chinese Patients with Chronic Diseases: A Population-Based Study. J. Affect. Disord..

[8] McGrath J.J., Lim C.C.W., Saha S. (2024). Cat Ownership and Schizophrenia-Related Disorders and Psychotic-Like Experiences: A Systematic Review and Meta-Analysis. Schizophr. Bull..

[9] O’Hanley K.A., Pearl D.L., Niel L. (2021). Risk Factors for Aggression in Adult Cats That Were Fostered through a Shelter Program as Kittens. Appl. Anim. Behav. Sci..

[10] Boonhoh W., Poolkhet C., Waran N., Wongtawan T. (2025). Systematic Review and Meta-Analysis of the Occurrence and Association of Physical Diseases and Behavioural Problems in Dogs and Cats. Appl. Anim. Behav. Sci..

[11] Ramos D., Mills D.S. (2009). Human Directed Aggression in Brazilian Domestic Cats: Owner Reported Prevalence, Contexts and Risk Factors. J. Feline Med. Surg..

[12] Stelow E.A., Bain M.J., Kass P.H. (2016). The Relationship Between Coat Color and Aggressive Behaviors in the Domestic Cat. J. Appl. Anim. Welf. Sci. JAAWS.

[13] Wassink-van der Schot A.A., Day C., Morton J.M., Rand J., Phillips C.J.C. (2016). Risk Factors for Behavior Problems in Cats Presented to an Australian Companion Animal Behavior Clinic. J. Vet. Behav..

[14] Amin O., Rostad C.A., Gonzalez M., Rostad B.S., Caltharp S., Quincer E., Betke B.A., Gottdenker N.L., Wilson J.J., Shane A.L. (2022). Cat Scratch Disease: 9 Years of Experience at a Pediatric Center. Open Forum Infect. Dis..

[15] Fehlner-Gardiner C., Gongal G., Tenzin T., Sabeta C., De Benedictis P., Rocha S.M., Vargas A., Cediel-Becerra N., Gomez L.C., Maki J. (2024). Rabies in Cats-An Emerging Public Health Issue. Viruses.

[16] Liu Y., Liu L., Kang M., Zong Z. (2020). An Unhealing Wound and Subcutaneous Nodules Due to Sporothrix Globosa after a Cat Bite. PLoS Negl. Trop. Dis..

[17] Taetzsch S.J., Bertke A.S., Gruszynski K.R. (2018). Zoonotic Disease Transmission Associated with Feral Cats in a Metropolitan Area: A Geospatial Analysis. Zoonoses Public Health.

[18] Baquero O.S., Chiozzotto E.N., Garcia R.d.C.M., Amaku M., Ferreira F. (2017). Abandonment of Dogs and Cats: Public Opinions as Population Management Indicators. J. Appl. Anim. Welf. Sci. JAAWS.

[19] Fatjó J., Bowen J., García E., Calvo P., Rueda S., Amblás S., Lalanza J.F. (2015). Epidemiology of Dog and Cat Abandonment in Spain (2008–2013). Animals.

[20] Bain M., Stelow E. (2014). Feline Aggression Toward Family Members: A Guide for Practitioners. Vet. Clin. N. Am. Small Anim. Pract..

[21] Martin H., Choi J.E., Rodrigues A.R., Eshel N. (2025). Review: Dopamine, Serotonin, and the Translational Neuroscience of Aggression in Autism Spectrum Disorder. JAACAP Open.

[22] Jahn K., DePorter T., Seksel K. (2023). Human-Directed Aggression and Pica in a 1-Year-Old Cat, Which Worsened Following International Relocation. JFMS Open Rep..

[23] Kaur G., Voith V.L., Schmidt P.L. (2016). The Use of Fluoxetine by Veterinarians in Dogs and Cats: A Preliminary Survey. Vet. Rec. Open.

[24] Masson S., Metz D., Bleuer-Elsner S., Schwobthaler F. (2025). Retrospective Study on the use of Venlafaxine in 176 Cats Diagnosed With Behavioral Disorders. J. Vet. Behav..

[25] Tu A.Y., Bell E.C. (2023). Owner-Directed Aggression in a 2-Year-Old Male Neutered Domestic Shorthair Cat: A Case Report. J. Vet. Behav..

[26] Denenberg S., Dubé M.B. (2018). Tools for Managing Feline Problem Behaviours Psychoactive Medications. J. Feline Med. Surg..

[27] DiCiccio V.K., McClosky M.E. (2022). Fluoxetine-Induced Urinary Retention in a Cat. JFMS Open Rep..

[28] Pugh C.M., Sweeney J.T., Bloch C.P., Lee J.A., Johnson J.A., Hovda L.R. (2013). Selective Serotonin Reuptake Inhibitor (SSRI) Toxicosis in Cats: 33 Cases (2004–2010). J. Vet. Emerg. Crit. Care.

[29] Masataka N. (2024). Is Cannabidiol (CBD) Effective to Ease Separation Anxiety?. Heliyon.

[30] Wanapinit K., Niyom S., Suriyawongpongsa P., Khathatip S., Tancharoen K., Roytrakul S., Ploypetch S. (2025). Evaluation of Cannabidiol Oil’s Effects on Sedation, Behavioral Responses to Handling, and Nociceptive Thresholds in Healthy Cats. Animals.

[31] Weller J.E., Flint H.E., Hunt A.B.G., Ellerby Z., King T. (2024). Investigating the Effect a Single Dose of Cannabidiol Has on Measures of Stress in Cats When Being Transported in a Carrier and Meeting a Novel Person in an Unfamiliar Environment. Front. Vet. Sci..

[32] Zanelli G.R., Vieira G.B.M., Souza R.V.M., Aguiar A.J.d.A., Cassu R.N. (2024). Perioperative Analgesic and Sedative Effects of Cannabidiol in Cats Undergoing Ovariohysterectomy. Animals.

[33] Brunt T.M., Bossong M.G. (2022). The Neuropharmacology of Cannabinoid Receptor Ligands in Central Signaling Pathways. Eur. J. Neurosci..

[34] Gutierre E., Crosignani N., García-Carnelli C., di Mateo A., Recchi L. (2023). A Case Report of CBD and THC as Analgesic Therapy in a Cat with Chronic Osteoarthritic Pain. Vet. Med. Sci..

[35] Miranda-Cortés A., Mota-Rojas D., Crosignani-Outeda N., Casas-Alvarado A., Martínez-Burnes J., Olmos-Hernández A., Mora-Medina P., Verduzco-Mendoza A., Hernández-Ávalos I. (2022). The Role of Cannabinoids in Pain Modulation in Companion Animals. Front. Vet. Sci..

[36] Kogan L.R., Hellyer P.W., Robinson N.G. (2016). Consumers’ Perceptions of Hemp Productions for Animals. J. Am. Holist. Vet. Med. Assoc..

[37] Kogan L.R., Hellyer P.W., Silcox S., Schoenfeld-Tacher R. (2019). Canadian Dog Owners’ Use and Perceptions of Cannabis Products. Can. Vet. J. Rev. Vet. Can..

[38] Pinto J.S., Martel F. (2022). Effects of Cannabidiol on Appetite and Body Weight: A Systematic Review. Clin. Drug Investig..

[39] Spanagel R., Bilbao A. (2021). Approved Cannabinoids for Medical Purposes—Comparative Systematic Review and Meta-Analysis for Sleep and Appetite. Neuropharmacology.

[40] Hartmann A., Lisboa S.F., Sonego A.B., Coutinho D., Gomes F.V., Guimarães F.S. (2019). Cannabidiol Attenuates Aggressive Behavior Induced by Social Isolation in Mice: Involvement of 5-HT1A and CB1 Receptors. Prog. Neuropsychopharmacol. Biol. Psychiatry.

[41] Corsetti S., Borruso S., Malandrucco L., Spallucci V., Maragliano L., Perino R., D’Agostino P., Natoli E. (2021). *Cannabis sativa* L. May Reduce Aggressive Behaviour towards Humans in Shelter Dogs. Sci. Rep..

[42] Marliani G., Vaccari L., Cavallini D., Montesano C.S., Buonaiuto G., Accorsi P.A. (2024). Assessing the Effectiveness of Cannabidiol Additive Supplementation on Canine Behavior and Cortisol Levels. Heliyon.

[43] Huang Y.H., Flynn J.P. (1975). Unit Activities in the Hypothalamus and Midbrain during Stimulation of Hypothalamic Attack Sites. Brain Res..

[44] Haller J. (2013). The Neurobiology of Abnormal Manifestations of Aggression—A Review of Hypothalamic Mechanisms in Cats, Rodents, and Humans. Brain Res. Bull..

[45] Nassif J.B., Felthous A.R. (2022). Mapping the Neurocircuitry of Impulsive Aggression through the Pharmacologic Review of Anti-Impulsive Aggressive Agents. J. Forensic Sci..

[46] da Cunha-Bang S., Knudsen G.M. (2021). The Modulatory Role of Serotonin on Human Impulsive Aggression. Biol. Psychiatry.

[47] León M., Rosado B., García-Belenguer S., Chacón G., Villegas A., Palacio J. (2012). Assessment of Serotonin in Serum, Plasma, and Platelets of Aggressive Dogs. J. Vet. Behav..

[48] Rosado B., García-Belenguer S., León M., Chacón G., Villegas A., Palacio J. (2010). Blood Concentrations of Serotonin, Cortisol and Dehydroepiandrosterone in Aggressive Dogs. Appl. Anim. Behav. Sci..

[49] Shaikh M.B., Siegel A. (1990). GABA-Mediated Regulation of Feline Aggression Elicited from Midbrain Periaqueductal Gray. Brain Res..

[50] Abame M.A., He Y., Wu S., Xie Z., Zhang J., Gong X., Wu C., Shen J. (2021). Chronic Administration of Synthetic Cannabidiol Induces Antidepressant Effects Involving Modulation of Serotonin and Noradrenaline Levels in the Hippocampus. Neurosci. Lett..

[51] Campos A.C., De Paula Soares V., Carvalho M.C., Ferreira F.R., Vicente M.A., Brandão M.L., Zuardi A.W., Zangrossi H., Guimarães F.S. (2013). Involvement of Serotonin-Mediated Neurotransmission in the Dorsal Periaqueductal Gray Matter on Cannabidiol Chronic Effects in Panic-like Responses in Rats. Psychopharmacology.

[52] Sales A.J., Crestani C.C., Guimarães F.S., Joca S.R.L. (2018). Antidepressant-like Effect Induced by Cannabidiol Is Dependent on Brain Serotonin Levels. Prog. Neuropsychopharmacol. Biol. Psychiatry.

[53] Bakas T., van Nieuwenhuijzen P.S., Devenish S.O., McGregor I.S., Arnold J.C., Chebib M. (2017). The Direct Actions of Cannabidiol and 2-Arachidonoyl Glycerol at GABAA Receptors. Pharmacol. Res..

[54] Ruffolo G., Gaeta A., Cannata B., Pinzaglia C., Aronica E., Morano A., Cifelli P., Palma E. (2022). GABAergic Neurotransmission in Human Tissues Is Modulated by Cannabidiol. Life.

[55] Pretzsch C.M., Freyberg J., Voinescu B., Lythgoe D., Horder J., Mendez M.A., Wichers R., Ajram L., Ivin G., Heasman M. (2019). Effects of Cannabidiol on Brain Excitation and Inhibition Systems; a Randomised Placebo-Controlled Single Dose Trial during Magnetic Resonance Spectroscopy in Adults with and without Autism Spectrum Disorder. Neuropsychopharmacol. Off. Publ. Am. Coll. Neuropsychopharmacol..

[56] Curtis T.M. (2008). Human-Directed Aggression in the Cat. Vet. Clin. N. Am. Small Anim. Pract..

[57] Rodan I., Dowgray N., Carney H.C., Carozza E., Ellis S.L., Heath S., Niel L., St Denis K., Taylor S. (2022). 2022 AAFP/ISFM Cat Friendly Veterinary Interaction Guidelines: Approach and Handling Techniques. J. Feline Med. Surg..

[58] Beaver B.V. (2004). Fractious Cats and Feline Aggression. J. Feline Med. Surg..

[59] Huestis M.A., Solimini R., Pichini S., Pacifici R., Carlier J., Busardò F.P. (2019). Cannabidiol Adverse Effects and Toxicity. Curr. Neuropharmacol..

