# Supplementary material for: Owner-Directed Feline Aggression in Thailand: Characteristics, Associated Factors, and a Clinical Comparison of Treatments
Source: Life (Basel). 2026 Feb 10;16(2):307. doi: 10.3390/life16020307 (PMC12942625; doi:10.3390/life16020307)
Supplement: Supplementary file 1 [file life-16-00307-s001.zip › life-4111792-supplementary.pdf]

Table S1 Questionnaire details for demographic data

**Owner information**	**Detail**
Gender	Man or Woman
Age (years)	Fill the age……
Ownership experience (years)	Fill the year…….
**Cat information**	
Sex	Male or Female
Reproductive Status	Intact or Neuter
Breeds	Fill the breed's name……….
**Environment information**	
House type	House or Condo
Living space	Indoor, Semi-indoor, or Outdoor
Number of humans in the household	
The presence of kids in the household (age < 18 years old)	Yes or No
Number of cats in the household (cat)	Fill the number……
The presence of other animals	Fill animal species……..
**Management**	
Food	Commercial or Cooked
Number of meals	Fill no. of meal……..
Method of providing water for the cat	Bowl or flowing water
Vaccinationn status	Annually or Not regularly
Body condition score	Fill body score 1-9…..
Number of litter boxes per cat	Fill no of litter of boxes……
Using catnips	Often or Rare
The presence scratcher	Yes or No
Climbing apparatus	Yes or No
**Owner-cat interaction**	
Reward-based training	Often or Rare
Punishment-based training	Often or Rare
Hug/Hold	Often or Rare
Head and chin petting	Often or Rare
Abdomen petting	Often or Rare
Kissing	Often or Rare
Traveling with a cat	Often or Rare
Sleeping behavior	With owner or not with the owner
The amount of time the cat is left alone at home daily	Fill the time……..

Table S2 Owner demographics, and their association with aggression score (n = 100).

**Category**	**Number**	**Percentage**	**Median of Aggression score**	**Association to aggression score**	
				**P-value**	**Statistics**
**Gender**				0.94	Man-Whitney
Man	20	20.00	2.25		-
Woman	80	80.00	2.37		-
**Age (years)**				0.25	Kruskal-Wallis
20-30	36	36.00	2.25		

31-40	32	32.00	2.50		
≥ 41	22	22.00	2.50		
**Ownership experience (years)**				0.88	Man-Whitney
≤ 5	55	55.00	2.38		
> 5	45	45.00	2.38		

Table S3 Cat demographics, and their association with aggression score (n = 100).

**Category**	**Number**	**Percentage**	**Median of Aggression score**	**Association to aggression score**	
				**P-value**	**Statistics**
**Sex**				0.56	Man-Whitney
Female	49	49.00	2.38		
Male	51	51.00	2.38		
**Age**				0.43	Man-Whitney
1-6 years old	91	91.00	2.38		
≥ 7 years old	9	9.00	2.38		
**Reproductive Status**				0.96	Man-Whitney
Intact	34	34.00	2.38		
Neuter	66	66.00	2.38		
**Breeds**				0.02*	Man-Whitney
Pure breed (pedigree)	26	26.00	2.62		
Mixed breed/Unknown/Native	74	74.00	2.31		

*Represent significant association ($P < 0.05$).

Table S4 Environmental characteristics, and their association with aggression score (n = 100).

**Category**	**Number**	**Percentage**	**Median of Aggression score**	**Association to aggression score**	
				**P value**	**Statistics**
**House**				0.83	Man-Whitney
House	80	80.00	2.37		
Dorm/Condo/Apartment	20	20.00	2.43		
**Living space**				0.36	Man-Whitney
Indoor	74	73.75	2.37		
Semi-indoor/outdoor	26	22.50	2.43		
**Number of humans in the household**				0.98	Kruskal-Wallis
1	15	15.00	2.50		
2-4	67	67.00	2.38		
> 4	18	18.00	2.38		
**The presence of kids in the household (age < 18 years old)**				0.43	Man-Whitney
Yes	24	24.00	2.38		

No	76	76.00	2.38		
**Number of cats in the household**				0.46	Kruskal-Wallis
1	35	35.00	2.25		
2	23	23.00	2.38		
≥ 3	42	42.00	2.38		
**The presence of other animals**				0.81	Man-Whitney
Yes	35	35.00	2.38		
No	65	65.00	2.38		

Table S5. Cat management practices by owners, and association with aggression score (n = 100).

Category	Number	Percentage	Median of Aggression score	Association to aggression score	
				P value	Statistics
**Food**				0.50	Man-Whitney
Commercial	95	95.00	2.37		
Homemade	5	5.00	2.70		
**Number of meals**				0.24	Man-Whitney
> 4 or All day	57	57.00	2.50		
1-4 times/day	43	43.00	2.25		
**Method of providing water**				0.81	Man-Whitney
Bowl	74ǂ	74.00	2.37		
Multiple methods (Bowl/Fountain/flowing water	26	26.00	2.43		
**Vaccinationn status**				0.45	Man-Whitney
Never or some year	40	40.00	2.38		
Annual	60	60.00	2.38		
**Body condition score**				0.64	Kruskal-Wallis
1-3 (underweight**)**	9	9.00	2.50		
4-6 (optimal)	61	61.00	2.37		
7-9 (overweight)	30	30.00	2.31		
**Number of litter boxes per cat**				0.09	Man-Whitney
Equal or higher than number of cats	62	62.00	2.37		
Less than number of cats	38	38.00	2.50		
**Using catnips**				0.99	Man-Whitney
Never/rare	92	92.00	2.38		
Often	8	8.00	2.38		
**The presence scratcher**				0.06	Man-Whitney
Yes	82	82.00	2.38		
No	18	18.00	2.50		
**Climbing apparatus**				0.96	Man-Whitney
Yes	55	55.00	2.38		
No	45	45.00	2.38		

Table S6 Human-cat interactions, and their association with aggression score (n = 100).

Category	Number	Percentage	Median of Aggression score	Association to aggression score	
				P value	Statistics
**Reward-based training**				0.03*	Man-Whitney
Never/Rare	22	22.00	2.75		
Often	78	78.00	2.25		

**Punishment-based training**				0.15	Man-Whitney
Never/Rare	77	77.00	2.37		
Often	23	23.00	2.25		
**Hug/Hold**				0.03*	Man-Whitney
Never/Rare	31	31.00	2.50		
Often	69	69.00	2.37		
**Head and chin petting**				0.03*	Man-Whitney
Never/Rare	25	25.00	2.50		
Often	75	75.00	2.37		
**Abdomen petting**				0.02*	Man-Whitney
Never/Rare	32ǂ	32.00	2.56		
Often	68	68.00	2.31		
**Kissing**				0.004*	Man-Whitney
Never/ Rare	42	42.00	2.71		
Often	58	58.00	2.25		
**Traveling with a cat**				0.69	Man-Whitney
Never/Rare	77	77.00	2.37		
Often	23	23.00	2.25		
**Stay with cat when feeding**				0.76	Man-Whitney
Never/Rare	47	47.00	2.38		
Often	53	53.00	2.38		
**Sleeping behavior**				0.18	Man-Whitney
Sleeping with the owner	63	63.00	2.37		
Not sleeping with the owner	37	37.00	2.50		
**The amount of time the cat is left alone at home daily**				0.47	Man-Whitney
≤ 4 hours	54	54.00	2.37		
> 4 hours	46	46.00	2.43		

*Represent significant association ($P < 0.05$).
